# Cesarean delivery in Norwegian nulliparous women with singleton cephalic term births, 1967–2020: a population-based study

**DOI:** 10.1186/s12884-022-04755-3

**Published:** 2022-05-18

**Authors:** Yeneabeba Tilahun Sima, Rolv Skjærven, Liv Grimstvedt Kvalvik, Nils-Halvdan Morken, Kari Klungsøyr, Linn Marie Sørbye

**Affiliations:** 1 Department of Global Public Health and Primary Care, University of Bergen, Bergen, Norway; 2 Department of Clinical Science, University of Bergen, Bergen, Norway; 3 Norwegian Research Centre for Women’s Health, Oslo University Hospital, Rikshospitalet, Oslo Norway

**Keywords:** Cesarean delivery, Population-based study, Robson groups, Norway

## Abstract

**Background:**

Nulliparous women contribute to increasing cesarean delivery in the Nordic countries and advanced maternal age has been suggested as responsible for rise in cesarean delivery rates in many developed countries. The aim was to describe changes in cesarean delivery rates among nulliparous women with singleton, cephalic, term births by change in sociodemographic factors across 50 years in Norway.

**Methods:**

We used data from the Medical Birth Registry of Norway and included 1 067 356 women delivering their first, singleton, cephalic, term birth between 1967 and 2020. Cesarean delivery was described by maternal age (5-year groups), onset of labor (spontaneous, induced and pre-labor CD), and time periods: 1967–1982, 1983–1998 and 1999–2020. We combined women’s age, onset of labor and time period into a compound variable, using women of 20–24 years, with spontaneous labor onset during 1967–1982 as reference. Multivariable regression models were used to estimate adjusted relative risk (ARR) of cesarean delivery with 95% confidence interval (CI).

**Results:**

Overall cesarean delivery increased both in women with and without spontaneous onset of labor, with a slight decline in recent years. The increase was mainly found among women < 35 years while it was stable or decreased in women >  = 35 years. In women with spontaneous onset of labor, the ARR of CD in women >  = 40 years decreased from 14.2 (95% CI 12.4–16.3) in 1967–82 to 6.7 (95% CI 6.2–7.4) in 1999–2020 and from 7.0 (95% CI 6.4–7.8) to 5.0 (95% CI 4.7–5.2) in women aged 35–39 years, compared to the reference population. Despite the rise in induced onset of labor over time, the ARR of CD declined in induced women >  = 40 years from 17.6 (95% CI 14.4–21.4) to 13.4 (95% CI 12.5–14.3) while it was stable in women 35–39 years.

**Conclusion:**

Despite growing number of Norwegian women having their first birth at a higher age, the increase in cesarean delivery was found among women < 35 years, while it was stable or decreased in older women. The increase in cesarean delivery cannot be solely explained by the shift to an older population of first-time mothers.

**Supplementary Information:**

The online version contains supplementary material available at 10.1186/s12884-022-04755-3.

## Introduction

Cesarean delivery (CD) has increased in all developed countries with Nordic countries having the lowest rates [1]. There has been a moderate increase in CD rates also in the Nordic countries [2]. Between 2000 and 2011 the rates increased by 26%, 15% and 10% in Denmark, Norway, and Sweden, respectively, after which they have levelled off during the most recent years [3]. Higher CD rates may be associated with future adverse complications in the mother and her baby [4] and have economic costs for the society [5].

The ongoing changes in clinical interventions as well as society composition including maternal age at childbirth and cultural background or ethnicity in relation to immigration, make it crucial to monitor CD rates and identify groups with too high CD rates and contributing factors [1]. Nulliparous women and women with previous CD are the two groups contributing strongly to increasing CD in the Nordic countries [2]. Major risk factors for the rise in CD include advanced maternal age [6], change in clinical practice such as management of breech pregnancies [7] and more induced deliveries [8]. Women’s preferences [9] and change in population risk profile such as higher body mass index (BMI) [10] are also important.

Increasing maternal age is associated with increased risk of pregnancy complications and obstructed labor [6], and may be explained by biological changes to the uterine contractility [11, 12]. However, a prior study among low risk nulliparous women in Norway and Sweden reported declining CD rates in women older than 35 years [13]. This study only focused on women older than 30 years and thus excluded most nulliparous women and did not take into consideration women’s different risk profiles and clinical handling.

Other factors influencing CD rates include changes in induction policy and pre-labor CD [8, 14]. The link between induction and CD has been much debated, with many studies reporting conflicting findings. Some observational studies report induction of labor in low-risk nulliparous women to increase risk of CD [15, 16] while others have reported unchanged or even lower risk of CD [17–19]. In Norway, induction rates have increased from 12.5% in 2003 to 20.3% in 2013, with one in ten inductions performed without any medical indication [20]. In 2020, the induction rate in Norway was 27.1% [21].

To address heterogeneity in risk of CD, the Robson classification has been used as a framework for comparing CD rates between groups with similar, clinically relevant risk factors for CD [22]. Robson groups R1 and R2 include nulliparous women with singleton, cephalic and term pregnancy, covering majority of nulliparous reproductive women [21]. The aim of our study was to describe changes in CD rates among these groups in relation to change in clinical intervention and sociodemographic factors in Norway across 50 years.

## Methods

### Data sources

In this population-based cohort study we analyzed data from the Medical Birth Registry of Norway (MBRN) between 1967 and mid-2020. The MBRN is based on mandatory notification of all live- and stillbirths from 16 weeks of gestation since 1967 [23] and prospectively collects data on mother’s health before and during pregnancy, as well as complications during and after delivery until discharge. Attending midwives or physicians are responsible for providing information to the registry. Before 1998, information was based on free text descriptions, which were coded using the International Classification of Diseases (ICD), 8th version. After 1998, checkboxes were introduced in addition to free text, and ICD-10 was used for coding. Information on maternal smoking habits was included in the MBRN in 1999, and mother’s height and weight gradually introduced from 2007. Data from the MBRN was linked to the Country-of-Origin Database and the National Education Database at Statistics Norway.

### Robson classification

We used the Robson classification to identify the study population [22]. This tool stratifies women based on five obstetric parameters: number of fetuses, fetal presentation, gestational age, previous CD, and onset of labor. Our study population included nulliparous women with singleton, cephalic, term birth with onset of labor as either spontaneous (Robson group R1), induced (Robson group R2a) or pre-labor CD (Robson group R2b). Similarly, to account for the acknowledged increased risk of CD in complicated pregnancies, separate analysis was done after excluding women with complications in their first pregnancy/delivery. Due to no direct information on indication for CD, we used the following complications as proxy for the indication: diabetes mellitus (before or during pregnancy), hypertension (chronic or during pregnancy), preeclampsia, post-term (> = 42 weeks), premature rupture of membranes (membrane rupture for > 24 h and unspecified time), placental abruption and placenta previa [21]. We adopted this potential indication list from the recent national Norwegian clinical guideline, provided by the Norwegian Society of Gynecology and Obstetrics [8].

### Study population

**Fig. 1:**
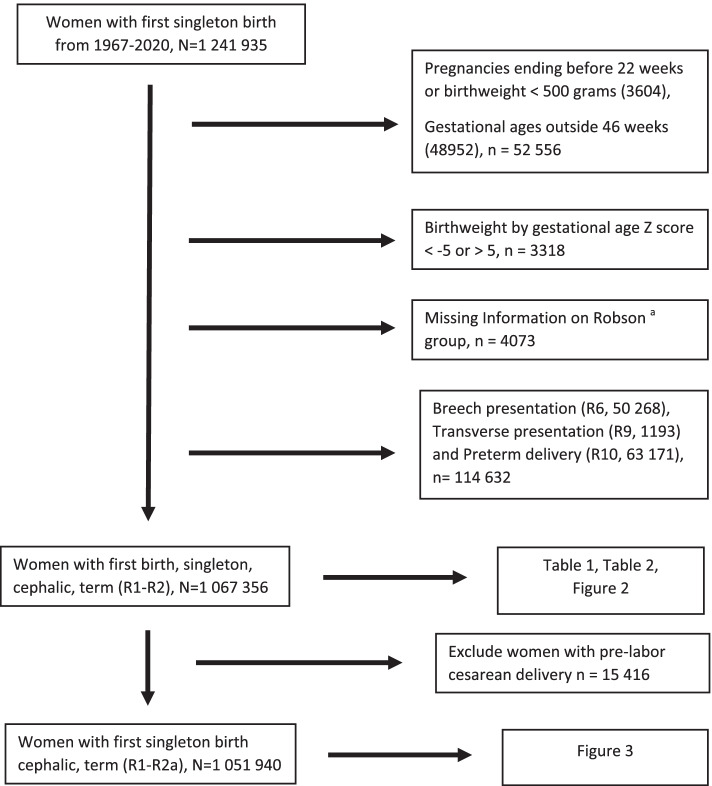
Flowchart of our study population. **a** Robson group stratifies women based on five obstetric parameters: number of fetuses, fetal presentation, gestational age, previous cesarean delivery and onset of labor

The study population included women who gave birth to their first singleton baby between 1967 and mid-2020. We excluded women with pregnancies ending before 22 weeks’ or infants weighting below 500 g, gestational ages outside of 46 completed weeks, infant’s birthweight by gestational age Z score [24] less than -5 or greater than 5 and women with missing information on Robson classification. Women in the other Robson groups (breech presentation (R6), transverse presentation (R9) and preterm delivery (R10)) were also excluded in order to have a homogenous population of nulliparous woman which makes up the majority of women of reproductive age. The final study population included women with singleton, cephalic term birth (Fig. 1).

### Cesarean delivery (CD)

CD was the outcome variable and proportions (CD rates) were calculated by dividing the number of CD by the number of deliveries during the specific period per 100 births. We showed the changes in CD over 50 years period. In addition, to capture changes in reporting format and obstetric practices across decades, we divided the years of delivery into three time periods: (1967–1982), (1983–1998) and (1999- 2020).

### Statistical analysis

Frequency and contingency tables were used to describe CD by maternal characteristics and onset of labor. Statistical analysis was carried out with STATA IC statistical software (version 16). Change in CD by onset of labor and maternal age groups (< 20, 20–24,25–29, 30–34, 35–39 and >  = 40) were assessed yearly and across three time periods, 1967–1982, 1983–1998 and 1999–2020. Generalized linear models with log link, binomial distribution and exponentiated regression coefficients were used to calculate adjusted relative risks (ARR) with 95% confidence intervals (CI) by periods. *P*-values below 0.05 were considered significant. A compound variable was made by combining maternal age, onset of labor (spontaneous (reference), induced and pre-labor CD) and time period, keeping women who had their first birth 20–24 years, with spontaneous labor onset in 1967–1982 as reference in the statistical model. Other variables included in the adjusted models were mother’s country of birth (Western women (reference): Europe, Canada, USA, New Zealand, and Australia, Non-western women: all other countries), offspring birthweight (continuous scale, in grams), smoking during pregnancy: (no (reference) and yes (daily/sometimes), restricted to births after 1999) and pregestational BMI (continuous scale, restricted to births after 2007). To test for linear CD trends within each maternal age category, we used year of delivery as a continuous variable. In addition, to evaluate the association between CD and maternal age (< 35, 35–39 and >  = 40) over time in relation to maternal education, we included an interaction term (Likelihood ratio test) between maternal age and maternal education (high: > 13 years (reference) and low: <  = 13 years). Associations were considered statistically significant at the 5% level.

## Results

A total of 1 067 356 nulliparous women with singleton, cephalic, term births were included. Table 1 shows sociodemographic changes across the three time periods. The proportion of women having their first birth >  = 35 years increased from 1.6% in 1967–1982 to 9.2% in 1999–2020. From first to last period, the proportion of women with > 13 years education more than doubled (from 26% to 58.7%) while the proportion of non-western women increased from 0.5% to 10.5%. The proportion of women with any of the seven pregnancy/delivery complications increased slightly, from 23.6% (1967–82) to 27.4% (1999–2020). The seven complications associated with CD were post-term (153,747, 14.4%), premature rupture of membrane (52,678, 4.9%), preeclampsia (38,362, 3.6%), chronic or gestational hypertension (23,302, 2.2%), pregestational or gestational diabetes mellitus (14,191, 1.3%), placental abruption (2706, 0.3%) and placenta previa (1170, 0.1%).

**Table 1:** Maternal characteristics at first singleton, cephalic, term birth, by three time periods in Norway, The Medical Birth Registry of Norway, 1967–2020, *N* = 1 067 356

Time period	1967–1982		1983–1998		1999–2020	
	N	%	N	%	N	%
Maternal age (years)						
< 20	66 043	20.1	27 505	9.0	17 639	4.1
20–24	163 585	49.8	114 526	37.5	97 448	22.5
25–29	76 728	23.3	113 394	37.2	168 214	38.8
30–34	17 184	5.2	39 245	12.9	110 421	25.5
35–39	4264	1.3	9198	3.0	33 832	7.8
> = 40	922	0.3	1210	0.4	5998	1.4
Maternal education						
Low (≤ 13 years)	241 227	73.4	171 373	56.2	165 305	38.1
High (> 13 years)	85 388	26.0	131 503	43.1	254 536	58.7
Missing	2211	0.7	2202	0.7	13 711	3.2
Maternal country of birth						
Western women	254 926	77.5	270 875	88.8	383 222	88.4
Non-western women	1771	0.5	10 546	3.5	45 689	10.5
Missing	72 029	21.9	23 657	7.8	4641	1.1
Pregnancy complications						
No pregnancy complications	251 152	76.4	232 605	76.2	314 718	72.6
Any pregnancy complications^**a**^	77 574	23.6	72 473	23.8	118 834	27.4
Total	328 726	100.0	305 078	100.0	433 552	100.0

^a^Women with one or more of the seven complications: diabetes mellitus (before or during pregnancy), hypertension (before or during pregnancy), preeclampsia, post-term, premature rupture of membrane (membrane rupture for > 24 h and unspecified time), placental abruption and placenta previa

**Fig. 2:**
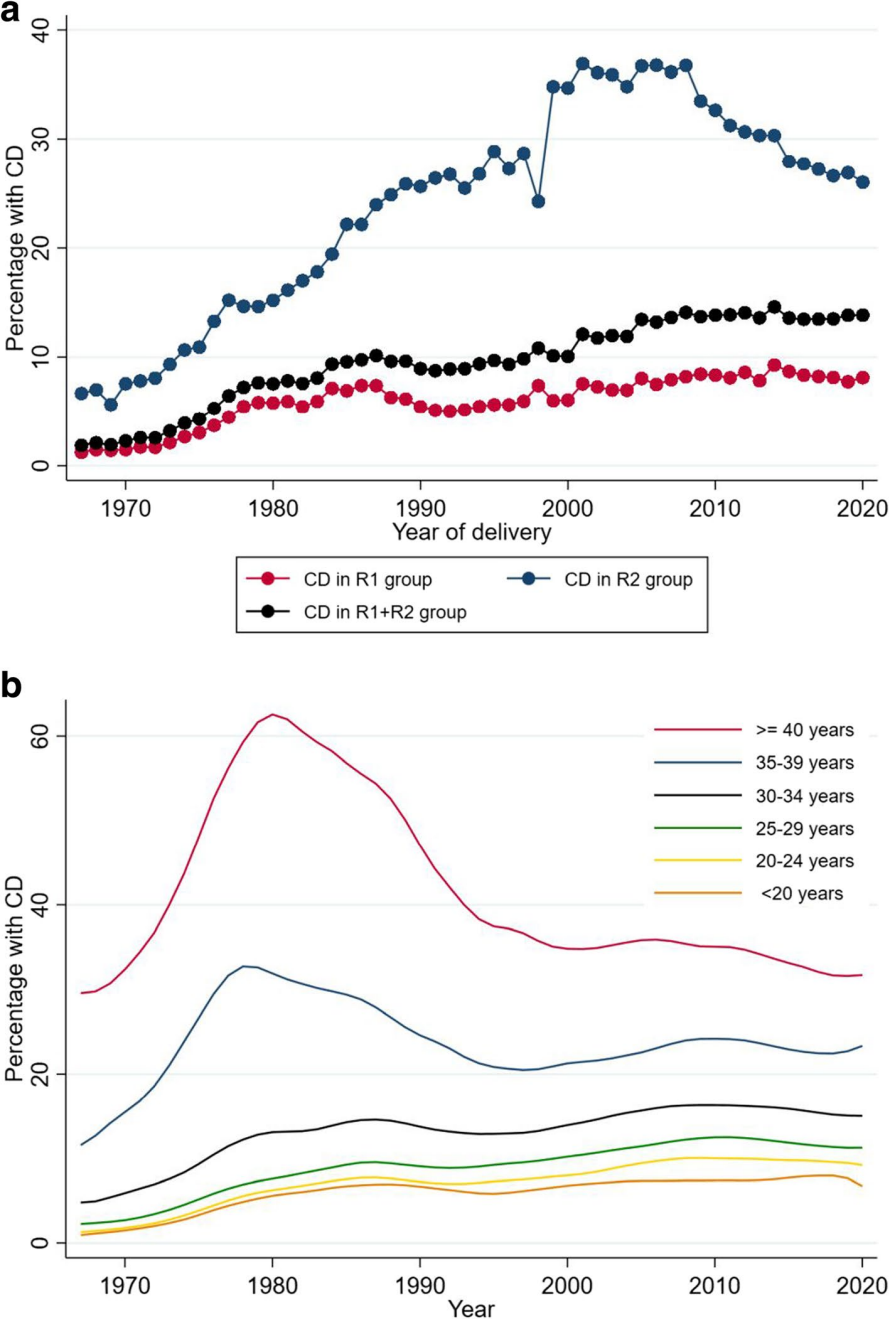
**a** The Proportion of cesarean delivery (CD) among nulliparous women with singleton, cephalic and term birth by onset of labor: spontaneous onset (R1) and those with induction onset and pre-labor cesarean delivery (R2), 1967–2020, *N* = 1 067 356. **b** The Proportion of cesarean delivery (CD) among nulliparous women with singleton, cephalic and term birth by maternal age, 1967–2020, *N* = 1 067 356

Overall CD increased, both in women with spontaneous onset of labor (R1) and those with either induction or pre-labor CD (R2) (Fig. 2a). There was a slight decline in CD in recent years, especially in the R2 group. In relation to the introduction of new reporting formats in 1999, the apparent change in the proportion of CD was limited to women in R2 group. CD increased with maternal age (Fig. 2b). The overall increase was mainly found among women < 35 years while it was stable or decreased in women >  = 35 years.

From first to last period, the proportion of women with term birth having spontaneous onset of labor declined, from 84.4 (1967–82) to 77.7% (1999–2020), while women having labor onset by induction or pre-labor CD increased, from 15.2% to 20.0% and from 0.4% to 2.4% respectively (Table S1). Women >  = 40 years had the highest decline in spontaneous onset of birth, from 71.0% (1967–82) to 47.4% (1999–2020), followed by women aged 35–39, from 74.0% (1967–82) to 66.7% (1999–2020). On the other hand, proportion of women with induced labor onset increased from 23.2% to 42.0% in women >  = 40 years and from 23.2 to 27.9% in women aged 35–39 years.

CD rates by onset of labor (spontaneous, induction and pre-labor CD), stratified by maternal age and time period, are presented in Table 2. The overall proportion of women having CD increased from 3.1% (1967–82) to 7.9% (1999–2020) and from 9.3% to 23.4% in the spontaneous onset- and induced onset group respectively. Among women with spontaneous onset of labor, CD increased in women < 35 years while it declined for women aged 35–39 years (from 18.3% to 13.3%) and for women above 40 years (35.0% to 17.5%). Similar changes in distribution across time and age groups were noted in women with induced onset of labor. For each respective maternal age group, proportion of CD was higher in women with onset of labor by induction than spontaneous labor, across all time periods. The contribution of pre-labor CD (R2b) to the group of women with induced or pre-labor CD (Robson R2) increased from 2.5% (1967–82) to 10.6% (1999–2020). This increment was found among women below 35 years while there was an inverse U form in women >  = 35 years.

**Table 2:** Cesarean delivery (CD) among nulliparous women with singleton, cephalic and term birth by onset of labor: spontaneous onset (R1), induction (R2a) and pre-labor cesarean delivery (R2b) and time period, *N* = 1 067 356

Time period	1967–1982		1983–1998		1999–2020	
	**n**	**CD (%)**^**a**^	**n**	**CD (%)**	**n**	**CD (%)**
Spontaneous onset (R1)						
< 20	57,484	3.5	23,403	4.5	14,725	4.6
20–24	139,406	2.4	96,312	5.0	79,317	5.9
25–29	63,139	3.7	93,270	6.1	133,935	7.3
30–34	13,571	7.2	30,746	8.6	83,395	9.5
35–39	3156	18.3	6551	14.7	22,576	13.3
> = 40	655	35.0	710	28.7	2844	17.5
Total	277,414	3.1	250,992	6.1	336,792	7.9
Onset by induction (R2a)						
< 20	8406	7.0	3886	13.2	2657	13.7
20–24	23,718	7.9	17,266	15.7	16,688	18.5
25–29	13,253	9.6	18,922	19.2	31,240	21.4
30–34	3459	14.8	7678	24.1	23,958	25.9
35–39	1000	30.8	2150	33.3	9427	31.2
> = 40	214	45.8	300	44.0	2520	35.7
Total	50,050	9.3	50,202	19.0	86,490	23.4
Pre-labor cesarean delivery (R2b/R2^c^)						
< 20	150	1.8	216	5.3	257	8.8
20–24	461	1.9	948	5.2	1443	8.0
25–29	336	2.5	1202	6.0	3039	8.9
30–34	154	4.3	821	9.7	3068	11.4
35–39	108	9.7	497	18.8	1829	16.2
> = 40	53	19.9	200	40.0	634	20.1
Total	1262	2.5	3884	7.2	10,270	10.6
All (R1 + R2a + R2b)						
< 20	66,043	2.9	27,505	6.5	17,639	7.3
20–24	163,585	3.5	114,526	7.4	97,448	9.4
25–29	76,728	5.1	113,394	9.3	168,214	11.6
30–34	17,184	9.5	39,245	13.6	110,421	15.6
35–39	4264	23.3	9198	23.7	33,832	23.0
> = 40	922	41.2	1210	44.3	5998	33.9
Total	328,726	4.4	305,078	9.4	433,552	13.1

^**a**^Total number of CD within the specific age group divided by total deliveries in the specific age group

^**c**^Summation of R2a and R2b

The sensitivity analysis, excluding women with any of the seven pregnancy/delivery complications, showed similar changes in CD over time and age groups for both the spontaneous—and induced onset groups. Within the group of women with either induced or pre-labor CD (R2), the proportion of pre-labor CD (R2b) was even higher after excluding women with complications, across all time periods. This shows that the increase in pre-labor CD over time was considerable among women without any of the seven pregnancy/delivery complications. Change in CD among nulliparous women in other Robson groups (breech (R6), transverse (R9) and preterm (R10)) is shown in Table S2.

**Fig. 3:**
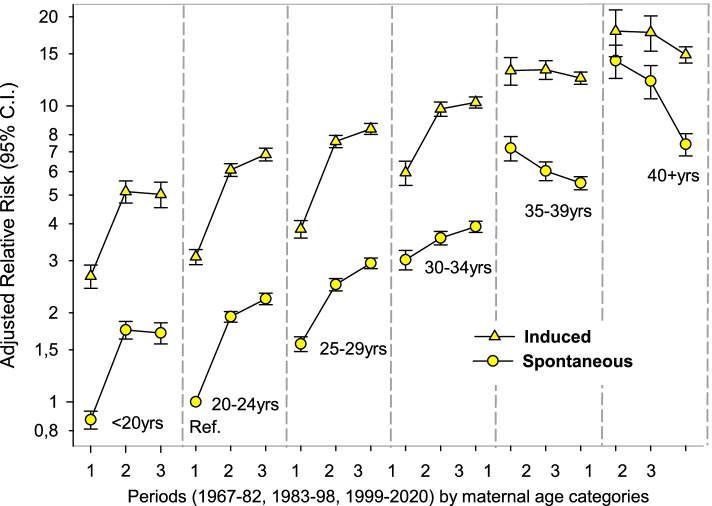
Adjusted Relative risk (ARR^**a**^) of cesarean delivery in nulliparous women with singleton, cephalic and term birth, stratified by maternal age, onset of labor: spontaneous onset, and induction onset, and time periods, *N* = 1 051 940. **a** Adjusted for maternal education, mother’s country of birth and birthweight

Compared to women 20–24 years with spontaneous onset of labor and giving birth in 1967–82, the ARR of CD increased across periods in all age groups < 35 years while it was stable or slightly decreased in women >  = 35 years (Fig. 3). ARR of CD in women >  = 40 years decreased from 14.2 (95% CI 12.4–16.3) in 1967–82 to 6.7 (95% CI 6.2–7.4) in 1999–2020 in women with spontaneous labor onset and from 17.6 (95% CI 14.4–21.4) to 13.4 (95% CI 12.5–14.3) in those with induced onset. Except for women aged 35–39 with induced onset of labor, we found a linear trend in CD across all other maternal age groups (Table S3). Excluding women with any of the seven pregnancy/delivery complications did not change the CD trend across time and age groups. The ARR of CD was higher in women from non-western countries (1.7, 95% CI 1.70–1.73). There was an interaction between the effect of maternal age and education on the risk of CD (Likelihood-ratio test, *p* < 0.001). Our main results stratified on maternal education are shown in Table S4. The gradual declining risk of CD among women >  = 35 years was more evident in those with high education than among those with low education. Results were similar after adjusting for smoking (restricted to births after 1999) and pre pregnancy BMI (restricted to births after 2007) (Table S5).

## Discussion

Overall CD increased over time in nulliparous women with singleton, cephalic and term birth. The increment was mainly observed among women < 35 years, while it was stable or decreased in women >  = 35 years. Although there has been increase in induction, risk of CD among women with induced labor decreased over time in women >  = 40 years, while it was stable in women 35–39 years. On the contrary, induction was associated with more CD over time in younger women.

Our study focused on nulliparous women with singleton, cephalic and term births. These women account for 90% of nulliparous and 40% of all reproductive women in Norway [21]. The proportion of women aged >  = 35 years at their first birth increased by time which is consistent to trends in other developed nations [11, 13, 15, 25]. Despite the growing number of Norwegian nulliparous women having their birth at a higher age, the increase in CD over time was found mainly among women < 35 years, while it was stable or reduced in older women. As advanced maternal age is strongly associated with higher risk of intrapartum CD due to higher prevalence of pregnancy complications [6] and biological changes in uterine contractility [11, 12], we expect a higher CD risk in the population of women >  = 35 years in the last period. However, since first time delivery at advanced age was less frequent in the first period of our study, it may be that clinicians more often viewed advanced age in nulliparous women as an independent indication for CD in the first time period than in the last. This could explain the stable/decreasing trend in CD among older women. The occurrence of the seven pregnancy complications increase with maternal age [6], which in turn is associated with increased risk of CD [26]. However, excluding these women from our analyses did not change CD trend across time or age groups.

Change in women’s preferences has been found to be another factor contributing to increased CD [9]. We found an increase in the proportion of R2b/R2 in the last relative to the first period and mainly among women < 35 years. This change over time was in fact larger in women without the common indications for CD. This increment could therefore not be explained by the studied pregnancy/delivery complications or other well-known obstetric indications, as we have excluded preterm, breech and multifetal pregnancies from our study population. It could be due to increased fear of giving birth or that women request CD for other reasons, without any evident medical or pregnancy complications [27]. An increase over time in other complications not captured by our list may also contribute some of this increment. A study from eight high income countries revealed knowledge gap as well as misconceptions about childbirth was more frequent in women who requested CD [9]. One out of 10 Norwegian women seemed to request CD with fear of pain, physical damages, and fear of insufficient support during delivery [28]. The recent increment in overweight and obesity in Norway, may also increase CD rates for all women [21]. For the years 2007–2020, we found the prevalence of overweight and obesity to be higher in nulliparous women aged >  = 35 years than younger women, similar to the findings from Denmark [29].

Despite the demographic changes to women’s age at first birth, CD declined over time among nulliparous Norwegian women >  = 35 years. This reduction suggests an important scope with tackling higher CD rates in other countries. The general less medicalized approach to childbirth in the Nordic countries where majority of births are attained by midwives [2], could explain the low CD rates in Norway compared to other developed countries [15, 25]. The national recommendations regarding induction of labor in women versus expectant management of labor [8, 14] may also explain the gradual decline of CD rates for women >  = 35 years.

On the relation between induction and CD, a recent Cochrane review on management of labor in women with term pregnancy found fewer CD in the induced group than those waiting for spontaneous onset of labor [14], in line with other studies [17–19]. In our study, the risk of CD was higher in women with induced than spontaneous onset of labor. We found that one out of five women with induced onset of labor had CD in 1999–2020, similar to a recent hospital based Norwegian study [26]. And only 8% of women with spontaneous onset of labor had CD in this period. Similarly, Ehrenthal et.al 2010 [16] and Davey et.al 2016 [15], reported higher risk of CD following induction in nulliparous women with term birth. Bergholt and colleagues reported that for every five-year increase in women’s age, the risk of CD increased 3 to 5 times for women with induced labor [30]. Despite the increase in induction among women >  = 40 years during our study period, the risk of CD declined in this age group. It could be argued that a more effective surveillance of labor with adherence to obstetric evidence-based practice could explain the decline in CD for this group [8, 14]. Besides women who have their first birth at advanced age are usually educated and with better socioeconomic support and with less risk factors such as smoking and overweight [31]. Declining CD rates among women > 35 were also reported in Sweden [13] and Canada [25].

A shift where CD is becoming more common among relatively younger nulliparous women should be concerning. The outcome of first pregnancy may affect women’s further reproduction including CD recurrence [4]. This is especially the case for countries where having two or more children is common, like Norway [21]. Hence it is important to keep the CD rate low among all nulliparous women, and especially in the younger women without complications. Policy makers and clinicians need to adapt measures that aim at lowering CD in first-time mothers, especially in women with low education and from non-western countries. Future research assessing the impact of current CD trends on long-term women’s health and reproduction is recommended.

### Strength and limitations

Strengths of this study are the large sample size, the comprehensive prospective population follow-up over almost five decades, which make both selection bias and recall bias less likely. In addition, missing data were low for most variables (< below 4%), except for country of birth during 1967–82. However, missing values for country of birth were evenly distributed by maternal age and education. Also, immigration to Norway during these years was low [21].

The study inherently has some limitations. Lack of data on the clinical indications for CD was handled by using pregnancy complications as a proxy for CD indication [26, 32]. We did, however, not have information on the two most common indications for CD, fetal distress and failure to progress [32]. Instead, we identified pregnancy complications that increase risk of both these two common indications. Changes in the reporting format in the MBRN is another limitation. Unlike checkboxes, notification based on free text may be linked to underreporting, especially of less severe complications [33] and a 3% error rate in completeness of CD notification for the years before 1984 has been reported [34]. This will likely have biased the result towards the null. Likewise, validity of data on initiated onset of labor (induced or pre-labor CD) was poor before the mid-1980s [35]. The findings after 1999 offer more precise and valid results. It’s however important to highlight that there have been several changes in clinical practice and sociodemographic factors within the last period. Our findings may have also underestimated changes for women without the seven pregnancy complications as complications may have been underreported in the early years of the MBRN [23]. Some women assumed to be without complications in the early period may in fact have been with complications. However, this means that the true increase in CD in women without seven complications is likely larger than reported here. Data on smoking and BMI were only available after 1999 and 2007, respectively.

## Conclusion

Monitoring CD is crucial to identify groups and factors contributing to high rates. This study described long-term changes in CD among Norwegian nulliparous women with singleton, cephalic term birth using large population-based data across five decades. A growing number of women are having their first birth at a higher age in Norway. The increase in CD rates in nulliparous women was mainly found among women < 35 years while it was stable or decreased in women >  = 35 years. Despite the increase in induction among women >  = 35 years during our study period, the risk of CD decreased in women >  = 40 years while it was stable in women 35–39 years. The overall increase in CD rates cannot be explained solely by the shift in age of first-time mothers.

## Supplementary Information

**Additional file 1.**

## Data Availability

Data belongs to the Norwegian Institute of Public Health and are only available to researchers who have applied for the data at the Medical Birth Registry of Norway. Restrictions apply to the availability of the data according to the license from the Regional Committee on Medical and Health Research Ethics. Data are however available from the Medical Birth Registry of Norway upon reasonable request and with permission from the Norwegian Institute of Public Health, [Access to data—NIPH (fhi.no)]**.**

## Abbreviations

CD: Cesarean delivery
MBRN: Medical Birth Registry of Norway
ARR: Adjusted relative risk
CI: Confidence interval
BMI: Body mass index
