# Supplementary material for: Cesarean delivery in Norwegian nulliparous women with singleton cephalic term births, 1967–2020: a population-based study
Source: BMC Pregnancy Childbirth. 2022 May 18;22:419. doi: 10.1186/s12884-022-04755-3 (PMC9118652; doi:10.1186/s12884-022-04755-3)
Supplement: Supplementary file 1 — Additional file 1. [file 12884_2022_4755_MOESM1_ESM.docx]

**Supplementary Information**

**Table S1.** The proportion of nulliparous women with singleton, cephalic, term birth by onset of labor: spontaneous onset (R1), induction (R2a) and pre-labor cesarean delivery (R2b), stratified by maternal age and time period, N=1 067 356

| **Time period** | **1967-1982** | | **1983-1998** | | **1999-2020** | |
| --- | --- | --- | --- | --- | --- | --- |
|  | **n** | **(%) ^a^** | **n** | **(%)** | **n** | **(%)** |
| Spontaneous onset (R1) |  |  |  |  |  |  |
| <20 | 57484 | 87 | 23403 | 85.1 | 14725 | 83.5 |
| 20-24 | 139406 | 85.2 | 96312 | 84.1 | 79317 | 81.4 |
| 25-29 | 63139 | 82.3 | 93270 | 82.3 | 133935 | 79.6 |
| 30-34 | 13571 | 79 | 30746 | 78.3 | 83395 | 75.5 |
| 35-39 | 3156 | 74 | 6551 | 71.2 | 22576 | 66.7 |
| >=40 | 655 | 71 | 710 | 58.7 | 2844 | 47.4 |
| total | 277414 | 84.4 | 250992 | 82.3 | 336792 | 77.7 |
| Onset by induction (R2a) |  |  |  |  |  |  |
| <20 | 8406 | 12.7 | 3886 | 14.1 | 2657 | 15.1 |
| 20-24 | 23718 | 14.5 | 17266 | 15.1 | 16688 | 17.1 |
| 25-29 | 13253 | 17.3 | 18922 | 16.7 | 31240 | 18.6 |
| 30-34 | 3459 | 20.1 | 7678 | 19.6 | 23958 | 21.7 |
| 35-39 | 1000 | 23.5 | 2150 | 23.4 | 9427 | 27.9 |
| >=40 | 214 | 23.2 | 300 | 24.8 | 2520 | 42 |
| total | 50050 | 15.2 | 50202 | 16.5 | 86490 | 20 |
| Pre-labor cesarean delivery (R2b/R2 ^b^) |  |  |  |  |  |  |
| <20 | 150 | 0.3 | 216 | 0.8 | 257 | 1.5 |
| 20-24 | 461 | 0.3 | 948 | 0.8 | 1443 | 1.5 |
| 25-29 | 336 | 0.4 | 1202 | 1.1 | 3039 | 1.8 |
| 30-34 | 154 | 0.9 | 821 | 2.1 | 3068 | 2.8 |
| 35-39 | 108 | 2.5 | 497 | 5.4 | 1829 | 5.4 |
| >=40 | 53 | 5.8 | 200 | 16.5 | 634 | 10.6 |
| total | 1262 | 0.4 | 3884 | 1.3 | 10270 | 2.4 |
| **Total** | 328 726 | 100.0 | 305 078 | 100.0 | 433 552 | 100.0 |

**^a^** Number of women within the specific R group divided by total women in the specific age group.

**^b^** Summation of R2a and R2b

**Table S2:** Cesarean delivery (CD) among nulliparous women in the other Robson groups (Breech (R6), Transverse (R9) and Preterm (R10)) stratified by maternal age and time period

| **Time period** | **1967-1982** | | **1983-1998** | | **1999-2020** | |
| --- | --- | --- | --- | --- | --- | --- |
|  | **n** | **CD (%) ^a^** | **n** | **CD (%)** | **n** | **CD (%)** |
| Breech presentation (R6) |  |  |  |  |  |  |
| <20 | 2219 | 26,9 | 885 | 41,9 | 570 | 31,2 |
| 20-24 | 5771 | 17,2 | 4425 | 40,9 | 3879 | 41,9 |
| 25-29 | 3246 | 7,8 | 5818 | 33,4 | 8856 | 58,7 |
| 30-34 | 834 | 4,3 | 2375 | 22 | 7310 | 73,7 |
| 35-39 | 214 | 4,2 | 611 | 18 | 2576 | 77,8 |
| >=40 | 55 | 6,3 | 93 | 14,7 | 531 | 79 |
| Total | 12339 | 9,5 | 14207 | 30,8 | 23722 | 59,8 |
| Transverse Presentation (R9) |  |  |  |  |  |  |
| <20 | 27 | 47,9 | 15 | 29,1 | 12 | 22,9 |
| 20-24 | 61 | 27,1 | 66 | 31,1 | 79 | 41,8 |
| 25-29 | 45 | 12,8 | 87 | 25,3 | 198 | 62 |
| 30-34 | 24 | 6,3 | 57 | 17,3 | 242 | 76,3 |
| 35-39 | 4 | 2,2 | 23 | 12,2 | 164 | 85,6 |
| >=40 | 7 | 8 | 8 | 8 | 74 | 84,1 |
| Total | 168 | 12,7 | 256 | 20,6 | 769 | 66,6 |
| Preterm (R10) |  |  |  |  |  |  |
| <20 | 5031 | 23,1 | 2072 | 43,2 | 1325 | 33,6 |
| 20-24 | 8403 | 16,7 | 6729 | 44,6 | 5598 | 38,7 |
| 25-29 | 3882 | 9 | 6551 | 37,2 | 9307 | 53,8 |
| 30-34 | 1184 | 6,6 | 2655 | 29,5 | 6198 | 63,9 |
| 35-39 | 370 | 7,5 | 813 | 26,3 | 2295 | 66,3 |
| >=40 | 114 | 8,7 | 132 | 18,9 | 512 | 72,4 |
| Total | 18984 | 11,3 | 18952 | 36,4 | 25235 | 52,3 |

**^a^** Total number of CD within the specific age group divided by total deliveries in the specific age group

**Table S3.** Adjusted relative risk (ARR) of cesarean delivery among nulliparous women with singleton, cephalic, term birth by maternal age and time period, stratified on onset of labor, N=1 051 940

| **Variables** | **All women** | | **Excluding women with complications ^b^** | |
| --- | --- | --- | --- | --- |
|  | **Cesarean delivery by onset of labor ARR^a^ (95% CI)** | | **Cesarean delivery by onset of labor ARR^a^ (95% CI)** | |
|  | **Spontaneous onset (R1)** | **Onset by induction (R2a)** | **Spontaneous onset (R1)** | **Onset by induction (R2a)** |
| 1967-82 | 0.9 (0.8-0.9) | 2.7 (2.5-3.0) | 0.9 (0.8-0.9) | 3.4 (3.0-3.9) |
| <20 years 1983-99 | 1.8 (1.6-1.9) | 5.2 (4.7-5.6) | 1.8 (1.7-2.0) | 5.6 (4.9-6.4) |
| 1999-2020 | 1.7 (1.6-1.9) | 5.1 (4.6-5.6) | 1.9 (1.8-2.1) | 6.2 (5.3-7.3) |
| P for trend | 0.000**(↑)** | 0.000**(↑)** | 0.000**(↑)** | 0.000**(↑)** |
| 1967-82 | 1 (Ref) | 3.1 (2.9-3.3) | 1 (Ref) | 3.5 (3.2-3.8) |
| 20-24 years 1983-99 | 1.9 (1.8-2.0) | 6 (5.7-6.3) | 2.0 (1.9-2.2) | 6.3 (5.9-6.8) |
| 1999-2020 | 2.2 (2.1-2.3) | 6.7 (6.4-7.0) | 2.4 (2.2-2.5) | 7.8 (7.3-8.3) |
| P for trend | 0.000**(↑)** | 0.000**(↑)** | 0.000**(↑)** | 0.000**(↑)** |
| 1967-82 | 1.5 (1.4-1.6) | 3.7 (3.4-4.0) | 1.6 (1.4-1.7) | 4.1 (3.7-4.5) |
| 25-29 years 1983-99 | 2.4 (2.2-2.5) | 7.2 (6.8-7.5) | 2.5 (2.3-2.6) | 7.7 (7.2-8.2) |
| 1999-2020 | 2.7 (2.6-2.8) | 7.7 (7.4-8.0) | 2.9 (2.8-3.1) | 9 (8.4-9.5) |
| P for trend | 0.000**(↑)** | 0.000**(↑)** | 0.000**(↑)** | 0.000**(↑)** |
| 1967-82 | 2.9 (2.7-3.2) | 5.7 (5.2-6.3) | 3.1 (2.8-3.4) | 6.5 (5.6-7.4) |
| 30-34 years 1983-99 | 3.3 (3.2-3.5) | 9.1 (8.6-9.6) | 3.6 (3.4-3.9) | 10 (9.2-10.8) |
| 1999-2020 | 3.5 (3.4-3.7) | 9.3 (8.9-9.7) | 3.9 (3.7-4.1) | 11 (10.3-11.7) |
| P for trend | 0.000**(↑)** | 0.000**(↑)** | 0.000**(↑)** | 0.000**(↑)** |
| 1967-82 | 7.0 (6.4-7.8) | 12.9 (11.5-14.4) | 7.6 (6.7-8.6) | 15.8 (13.6-18.3) |
| 35-39 years 1983-99 | 5.6 (5.2-6.1) | 12.5 (11.6-13.4) | 6.3 (5.7-6.9) | 14.4 (12.9-13.4) |
| 1999-2020 | 5.0 (4.7-5.2) | 11.3 (10.8-11.8) | 5.6 (5.2-5.9) | 13.1 (12.3-11.8) |
| P for trend | 0.000**(↓)** | 0.000**(↓)** | 0.000**(↓)** | 0.27**(↓)** |
| 1967-82 | 14.2 (12.4-16.3) | 17.6 (14.4-21.4) | 16.7 (14.2-16.3) | 19.6 (14.8-25.8) |
| >=40 years 1983-99 | 11.4 (9.9-13.1) | 16.9 (14.7-19.5) | 13.2 (11.3-15.6) | 20.8 (17.2-25.1) |
| 1999-2020 | 6.7 (6.2-7.4) | 13.4 (12.5-14.3) | 7.6 (6.8-8.5) | 16.5 (15.1-18.0) |
| P for trend | 0.000**(↓)** | 0.000**(↓)** | 0.000**(↓)** | 0.000**(↓)** |
| Mother's country of birth |  |  |  |  |
| Western women | 1 | | 1 | |
| Nonwestern women | 1.7 (1.70-1.73) | | 1.83 (1.8-1.9) | |
| Birthweight ^c^ | 1.00 (1.00-1.00) | | 1.00 (1.00-1.00) | |

**^a^** adjusted for country of birth and birthweight

**^b^** Excluding women with any of the seven pregnancy complications (diabetes mellitus (before or during pregnancy), hypertension (before or during pregnancy), preeclampsia, post-term, premature rupture of membrane (membrane rupture for > 24 hour and unspecified time), placental abruption and placenta previa)

^c^ Modeled as a continuous, linear term

**(↑)**: increase in trend

**(↓)**: decrease in trend

**Table S4.** Adjusted relative risk (ARR) of cesarean delivery among nulliparous women with singleton, cephalic, term birth by maternal age and time period, stratified on onset of labor and maternal education, N=1 051 940

|  | **All women**  **ARR^a^ (95% CI)** | | | | **Spontaneous onset (R1)**  **ARR^a^ (95% CI)** | | | **Onset by induction (R2a)**  **ARR^a^ (95% CI)** | |
| --- | --- | --- | --- | --- | --- | --- | --- | --- | --- |
|  | Low **^b^**  education | | High **^c^**  education | | Low  education | | High  education | Low education | High education |
| 1967-82 | | 0.9 (0.8-0.9) | | 1.0 (Ref) | | 0.9 (0.8-0.9) | 1.0 (Ref) | 1.0 (0.9-1.1) | 1.0 (Ref) |
| <35years 1983-99 | | 1.9 (1.8-1.9) | | 1.8 (1.7-1.8) | | 1.9 (1.7-1.9) | 1.7 (1.6-1.8) | 2.1(2.0-2.3) | 2.0 (1.9-2.1) |
| 1999-2020 | | 2.4 (2.3-2.5) | | 2.2 (2.1-2.3) | | 2.3 (2.2-2.4) | 2.0 (1.9-2.1) | 2.5 (2.4-2.7) | 2.3 (2.2-2.5) |
| 1967-82 | | 4.9 (4.4-5.4) | | 6.0 (5.3-6.7) | | 5.3 (4.6-6.0) | 6.3 (5.4-7.3) | 3.5 (3.0-4.0) | 4.5 (3.8-5.3) |
| 35-39years 1983-99 | | 5.2 (4.8-5.6) | | 3.9 (3.7-4.3) | | 5.0 (4.5-5.6) | 4.0 (3.6-4.4) | 4.3 (3.9-6.4) | 3.3 (2.9-3.7) |
| 1999-2020 | | 4.7 (4.5-5.0) | | 3.8 (3.8-4.2) | | 4.4 (4.1-4.8) | 3.7 (3.5-3.9) | 3.8 (3.5-4.1) | 3.2 (3.0-3.5) |
| 1967-82 | | 8.6 (7.5-9.9) | | 11.3 (9.3-13.7) | | 10.5 (8.9-12.4) | 14.3 (11.2-18.3) | 5.1 (3.9-6.4) | 5.6 (4.0-7.7) |
| >=40 years 1983-99 | | 9.4 (8.2-10.8) | | 6.9 (5.9-8.1) | | 11.6 (9.7-13.8) | 7.1 (5.8-8.8) | 5.0 (4.1-6.2) | 5.1 (4.2-6.2) |
| 1999-2020 | | 6.6 (6.0-7.2) | | 5.9 (5.6-6.4) | | 6.2 (5.4-7.2) | 4.9 (4.4-5.5) | 4.2 (3.8-4.7) | 4.0 (3.7-4.3) |

**^a^** adjusted for country of birth and birthweight

**^b^** <=13 years

**^c^** > 13 years

**Table S5:** Adjusted relative risk (ARR) of cesarean delivery among nulliparous women with singleton, cephalic, term birth by smoking and pregestational body mass index, The Medical Birth Registry of Norway, 1999-2020

1. Smoking: The Medical Birth Registry of Norway, 2007-2020, n= 346 241

|  |  |  |
| --- | --- | --- |
| **Variables** | **Crude RR (95% CI)** | **ARR^a^ (95% CI)** |
| Maternal age (years) |  |  |
| <20 | 0.8 (0.7-0.8) | 0.7 (0.7-0.8) |
| 20-24 | Reference | Reference |
| 25-29 | 1.3 (1.2-1.3) | 1.3 (1.3-1.4) |
| 30-34 | 1.7 (1.6-1.7) | 1.7 (1.7-1.8) |
| 35-39 | 2.1 (2.1-2.2) | 2.2 (2.1-2.3) |
| >=40 | 2.7 (2.5-2.8) | 2.7 (2.6-2.9) |
| Year of delivery ^b^ | 1.00 (0.9-0.9) | 1.00 (0.9-0.9) |
| Onset of labor |  |  |
| Spontaneous onset (R1) | 1 | 1 |
| Induced onset (R2a) | 2.6 (2.6-2.7) | 2.6 (2.5-2.6) |
| Maternal Education |  |  |
| High (> 13 years) | 1 | 1 |
| Low (<=13 years) | 1.3 (1.2-1.3) | 1.2 (1.2-1.3) |
| Mother’s country of birth |  |  |
| Western women | 1 | 1 |
| Nonwestern women | 1.7 (1.6-1.7) | 1.8 (1.7-1.8) |
| Birthweight ^b^ | 1.00 (1.0-1.0) | 1.0 (1.0-1.0) |
| Smoking |  |  |
| No |  | 1 |
| daily/sometimes |  | - 1. (1.1-1.2) |

^a^ adjusted for smoking

^b^ Modeled as a continuous, linear term

1. Pregestational body mass index (BMI): The Medical Birth Registry of Norway, 2007-2020, n=181 148

| **Variables** | **Crude RR (95% CI)** | **ARR ^a^ (95% CI)** |
| --- | --- | --- |
| Maternal age (years) |  |  |
| <20 | 0.7 (0.6-0.8) | 0.7 (0.6-0.8) |
| 20-24 | Reference | Reference |
| 25-29 | 1.3 (1.3-1.4) | 1.3 (1.3-1.4) |
| 30-34 | 1.8 (1.7-1.8) | 1.8 (1.7-1.8) |
| 35-39 | 2.3 (2.2-2.4) | 2.2 (2.1-2.3) |
| >=40 | 2.7 (2.5-2.9) | 2.6 (2.4-2.8) |
| Year of delivery ^b^ | 0.99 (0.98-0.99) | 0.99 (0.98-0.99) |
| Onset of labor |  |  |
| Spontaneous onset (R1) | 1 | 1 |
| Induced onset (R2a) | 2.4 (2.3-2.5) | 2.2 (2.2-2.3) |
| Maternal Education |  |  |
| High (> 13 years) | 1 | 1 |
| Low (<=13 years) | 1.3 (1.2-1.3) | 1.2 (1.1-1.2) |
| Mother’s country of birth |  |  |
| Western women | 1 | 1 |
| Nonwestern women | 1.7 (1.7-1.8) | 1.8 (1.8-1.9) |
| Birthweight ^b^ | 1.00 (1.00-1.00) | 1.00 (1.00-1.00) |
| Smoking |  |  |
| No | 1 | 1 |
| daily/sometimes | 1.3 (1.2-1.4) | 1.2 (1.1-1.3) |
| BMI ^b^ |  | 1.04 (1.03-1.04) |

^a^ adjusted for BMI

^b^ Modeled as a continuous, linear term
